# Determinants of prolonged weaning-to-service interval in primiparous Landrace × Yorkshire sows under tropical conditions: Impact of age at first farrowing, lactation length, and litter size at weaning

**DOI:** 10.14202/vetworld.2025.2031-2038

**Published:** 2025-07-27

**Authors:** Nguyen Hoai Nam, Do Thi Kim Lanh, Nguyen Van Thanh, Bui Van Dung, Peerapol Sukon

**Affiliations:** 1 Department of Animal Surgery and Theriogenology, Faculty of Veterinary Medicine, Vietnam National University of Agriculture, Hanoi, Vietnam; 2 Department of Anatomy, Faculty of Veterinary Medicine, Khon Kaen University, 123 Moo 16 Mittraphap Rd., Nai-Muang, Muang, Khon Kaen, 40002, Thailand; 3 KKU Research Program, Khon Kaen University, 123 Moo 16 Mittraphap Rd., Nai-Muang, Muang, Khon Kaen, 40002, Thailand

**Keywords:** age at first farrowing, lactation length, litter size at weaning, primiparous sows, reproductive performance, tropical swine production, weaning-to-service interval

## Abstract

**Background and Aim:**

Primiparous sows are particularly vulnerable to prolonged weaning-to-service interval (WSI), which negatively impacts reproductive efficiency and farm profitability. This study aimed to identify critical risk factors associated with prolonged WSI (>6 days) in first-parity Landrace × Yorkshire sows raised under tropical conditions.

**Materials and Methods:**

A retrospective cohort analysis was performed using production records from 3,222 sows on a commercial farm in Central Vietnam. Data on age at first artificial insemination, age at first farrowing (AFF), lactation length (LL), litter size at weaning (LSW), number born alive (NBA), and litter birth weight (LBW) were analyzed. Univariate and multivariate logistic regression models were used to identify predictors of prolonged WSI.

**Results:**

Prolonged WSI was observed in 34.6% of sows. Multivariate analysis identified three significant predictors: (1) Early AFF (302–360 days) was associated with higher odds of prolonged WSI compared to older age groups (odds ratio [OR] range: 0.38–0.51, p < 0.001). (2) Short LL (12–22 days) increased WSI risk, while LL of 25–26 days had the lowest risk (OR = 0.39, p < 0.001). (3) Higher LSW (≥12 piglets) was positively associated with prolonged WSI (OR = 1.41–1.63, p < 0.05). NBA and LBW were not significantly associated with prolonged WSI.

**Conclusions:**

Early AFF, shorter LL, and larger LSW are key risk factors for prolonged WSI. Management practices that optimize gilt development (target AFF >360 days), extend lactation to ~25 days, and avoid excessive LSW (>11 piglets) may reduce WSI and enhance reproductive performance under tropical conditions.

## INTRODUCTION

Primiparous sows represent a significant propo-rtion of breeding herds worldwide [1–3]. Unlike multi- parous sows, they continue to grow during both gestation and their first lactation cycle [4–6]. As such, their nutritional and energy requirements must sup-port not only fetal development and milk production but also their own somatic growth [7, 8]. However, primiparous sows typically exhibit lower feed intake during lactation and experience greater losses in body weight, backfat, adipose tissue, and protein rese-rves compared to their multiparous counterparts [9]. These physiological challenges predispose them to reproductive inefficiencies, including second litter syndrome, reported in approximately 46%–57% of sows [10–12], and extended weaning-to-service inter-vals (WSI) [9, 13–15].

The WSI is a critical reproductive metric that influences overall productivity, especially under both tropical and temperate conditions. Prolonged WSI contributes substantially to non-productive days in sow herds [16]. Studies conducted in Japan and Thailand have demonstrated that WSI affects farrowing rates [17, 18], litter sizes [19, 20], and culling rates [21]. For instance, in tropical settings, sows inseminated within 0–6 days post-weaning had significantly higher farrowing rates than those mated after 7–20 days (86.8% vs. 78.9%) [17]. A similar pattern was observed under temperate climates, where farrowing rates dropped from 85% to 72% when insemination occurred after 6 days [18]. In Brazil, peak farrowing rates were recorded in sows bred 3–5 days post-weaning, regardless of parity status [22]. In addition, larger litter sizes and reduced culling rates were observed in sows served within 1–5 or 4–6 days post-weaning compared to those bred beyond day 6 or 7 in both Thai and Japanese herds [18, 20, 21].

Several risk factors associated with prolonged WSI have been extensively studied. Research from Australia, the United States, China, and the Netherlands indicates an inverse relationship between lactation length (LL) and WSI [15, 23–25], although some exce-ptions exist, such as a positive association reported in Brazilian herds [26]. Consistent findings from Thailand, Vietnam, Lithuania, and the Netherlands suggest that higher parity is associated with a reduction in WSI duration [13–14, 25, 27]. Contrasting results have been reported regarding litter size at weaning (LSW); some studies, such as those by Vesseur *et al*. [25] and Sterning *et al*. [28], describe a positive relationship with WSI, while a study by Alexopoulos *et al*. [29] reports a negative correlation. Moreover, excessive weight loss during lactation has been shown to delay the return to estrus and extend WSI [28, 30, 31].

Emerging data from Vietnam highlight a negative association between litter size at birth and WSI [27, 32], contradicting earlier findings from Sweden [28]. Other potential contributors to prolonged WSI include enviro-nmental and management-related factors such as housing systems [25], seasonal effects [14, 25], age at puberty [33], temperature-humidity index [33–35], gilt body weight at first mating [7], and nutritional strategies [8]. Reproductive parameters such as split suckling [36], manual farrowing assistance, stillbirth occurrence, vaginal discharge duration, and gestation length have also been explored [27]. Serenius *et al*. [37] have focused specifically on how the age at first insemination and age at first farrowing (AFF) influence WSI outcomes in primiparous sows.

Despite the extensive literature exploring factors influencing the WSI in sows, the majority of existing studies have focused on mixed-parity populations or multiparous sows, with limited attention given exclu- sively to primiparous sows – a group inherently different in physiological and metabolic profiles. Primiparous sows, due to their continued growth and immature reproductive systems, are more vulne-rable to postpartum reproductive disorders, including prolonged WSI. In addition, while variables such as LL, LSW, body weight changes, environmental stress, and nutritional management have been associated with WSI outcomes, the specific influence of early-life reproductive traits – particularly age at first artificial insemination (AFAI) and AFF – remains underexplored. These factors may be critical in shaping postpartum reproductive performance, especially under tropical climatic conditions where heat stress and seasonal variation further compromise sow fertility.

Moreover, previous findings on the effects of LSW and litter size at birth on WSI have been inconsistent and sometimes contradictory across geographic and environmental contexts. Some studies report posi-tive associations, while others suggest negative or no significant correlations. The majority of such work has been conducted in temperate climates, with limited data from tropical, high-humidity environments, which present additional physiological challenges. Thus, there is a significant need for region-specific, parity-specific, and climate-contextualized investigations that clarify the predictors of prolonged WSI in commercial sow operations.

This study aims to comprehensively evaluate the risk factors associated with prolonged WSI in prim-iparous Landrace × Yorkshire sows managed under tropical conditions in Central Vietnam. Specifically, it seeks to: (1) Quantify the effect of AFAI and AFF on the likelihood of prolonged WSI. (2) Assess the influence of LL and LSW on post-weaning reproductive recovery. (3) Identify optimal thresholds for key management parameters (e.g., LL and AFF) that may minimize the risk of prolonged WSI without compromising lifetime productivity.

By focusing on a large dataset from a commercial herd (n = 3,222 sows), this study aims to provide evidence-based recommendations to enhance repro-ductive efficiency, guide gilt development strategies, and optimize herd productivity in tropical production systems.

## MATERIALS AND METHODS

### Ethical approval

This retrospective study utilized existing farm production records and did not involve any direct interv-entions or experimental procedures involving animals. Therefore, formal ethical approval was not required. Nonetheless, all data collection procedures adhered to standard ethical guidelines for the use of animals in research.

### Study period and location

The study was conducted from August to Dece-mber 2024 on a commercial swine farm located in the central region of Vietnam, comprising approximately 5,000 crossbred Landrace × Yorkshire breeding sows. The sows included in the analysis were born between October 2022 and March 2023, with their first farrowing and lactation periods occurring between October 2023 and July 2024. The regional climate is characterized by an average annual temperature of 22°C–29°C and relative humidity of approximately 85%–86%.

### Herd health and breeding management

Before breeding, gilts were vaccinated accor-ding to standard protocols against porcine circovirus, *Mycoplasma hyopneumoniae*, foot-and-mouth disease, Aujeszky’s disease virus, porcine parvovirus, class-ical swine fever, *Erysipelothrix rhusiopathiae*, and *Esch-erichia coli*. Gilts were group-housed until puberty and inseminated during their second or third estrus once they reached a minimum body weight of 135 kg. Inseminations were conducted twice using fresh semen (≥75% motility; ≥3 billion sperm cells/dose).

Post-breeding, sows were revaccinated agai-nst key pathogens. During gestation, sows received 2.2–2.4 kg/day of a commercial diet containing ~14% crude protein and 15 MJ/kg metabolizable energy. During lactation, sows were fed *ad libitum* on a diet con-taining ~16% crude protein and similar energy levels. Feed allowances after weaning were 2.5–3.0 kg/day, adjusted according to body condition.

### Farrowing and lactation management

Natural farrowing was allowed; however, if sows did not farrow by day 116 of gestation, parturition was induced using 175 μg cloprostenol (Hanprost, Hanvet, Vietnam). Dystocic sows were assisted with oxytocin (20 IU, DonaVet, Vietnam) or manual extraction when necessary. Cloprostenol and oxytocin were administered through the perivulvar route.

Cross-fostering was conducted within the first 24 h after birth; split suckling was not performed. Prew-eaning mortality was approximately 7%. Sows were weaned after an average of 25 days, with 9% weaned before 21 days.

### Post-weaning management and estrus detection

Following weaning, sows received a vitamin A, D, and E injection (500,000 IU A, 250,000 IU D, 120 mg E; 6 mL; DonaVet, Vietnam) and were exposed to intact Meishan boars for a minimum of 6 h/day (1 boar/12 sows). Estrus detection was conducted once daily using the backpressure test in the presence of a boar.

Sows not exhibiting estrus within 7 days post-weaning received a second vitamin ADE injection. If estrus was still absent by day 14, a fasting regimen on alternate days was initiated. For sows not returning to estrus by days 18–21, hormonal induction was applied using 400 IU of equine chorionic gonadotropin and 200 IU of human chorionic gonadotropin (Heat 5×, Dong Bang Co., Ltd., Korea), administered intramuscularly into the neck. Lighting and boar exposure durations were increased to 12 and 16 h/day, respectively, in refractory cases.

### Data collection and variable definition

An Excel database (Microsoft Office 2016, Wash-ington, USA) containing records from 7,590 gilts and sows was provided by the farm. Of these, 3,222 primiparous sows had complete records on:

- Date of first artificial insemination
- Date of first farrowing
- Number born alive (NBA, piglets) Only live-born piglets with birth weights >0.85 kg were included, which may underestimate total live births and LBW.
- Litter birth weight (LBW, kg)
- Date of weaning
- LSW
- Date of first post-weaning service.

From these records, the following variables were computed:

- AFAI (days)
- AFF (days)
- LL (days)
- WSI (days).

Prolonged WSI was defined as >6 days based on classifications by Yatabe *et al*. [38] and Hoshino and Koketsu [18], which link longer intervals to reduced farrowing rates.

### Statistical analysis

All analyses were performed using IBM SPSS Statistics for Windows, Version 22.0 (IBM Corp., NY, USA). A two-stage logistic regression approach was employed:

1.Univariate analysis to assess associations between each independent variable and prolonged WSI.
2.Multivariate logistic regression using forward step-wise selection for variables meeting the threshold of p < 0.20.

Independent variables were categorized as follows:

- AFAI: 189–240, 241–250, 251–260, 261–270, 271–280, 280–436 days
- AFF: 302–360, 361–370, 371–380, 381–390, 391–400, 400–554 days
- NBA: <6, 6–10, 11–15, 16–21 piglets
- LBW: <10, 10–15, 15–18, >18 kg
- LL: 12–22, 23–24, 25–26, 27–28, >28 days
- LSW: <11, 11, 12, 13, 14–18 piglets.

Odds ratios (OR) with 95% confidence intervals (CI) were calculated. Variables with high multicollinearity (Spearman’s ρ ≥ 0.70) were excluded – AFAI was dropped due to its strong correlation with AFF (ρ = 0.97).

Model performance was evaluated using the Hosmer-Lemeshow goodness-of-fit test and Nagelkerke R^2^. Model selection was guided by the Akaike infor-mation criterion (AIC) and the Bayesian information criterion (BIC). The area under the ROC curve (AUC) was computed to assess discriminatory ability. Statistical significance was set at p < 0.05 (two-tailed).

## RESULTS

### Descriptive statistics

The average WSI among the 3,222 primiparous sows was 8.7 ± 8.0 days, with 34.6% (1,114/3,222) exhibiting a prolonged WSI (>6 days). The mean AFAI and AFF were 267.7 ± 32.2 days and 384.6 ± 32.3 days, respectively. The average LBW and LL were 16.0 ± 3.4 kg and 25.1 ± 3.4 days, respectively. The mean NBA and LSW were 12.6 ± 2.7 and 12.3 ± 1.5, respectively.

The incidence of prolonged WSI varied across AFAI groups, with the highest rate observed in sows inseminated between 189 and 240 days (56.7%, 242/427). This proportion decreased to 40.8% in the 241–250 day group (156/382) and ranged from 28.3% to 31.9% in those inseminated after 250 days.

A similar trend was observed for AFF, where the youngest group (302–360 days) had the highest incidence of prolonged WSI (55.0%, 298/542). This rate decreased progressively in older age groups, with the lowest incidence recorded in the 381–390 day group (26.4%, 174/660).

No clear pattern was evident across the NBA or LBW categories. The incidence of prolonged WSI across NBA groups ranged from 31.0% (18/58) to 39.1% (133/340), and for LBW groups, from 32.0% (425/1,328) to 39.1% (261/667).

In contrast, LL was inversely associated with prolonged WSI. Sows with a short LL of 12–22 days had the highest incidence (53.7%, 376/700), which decreased to 34.0% (201/592) in those with a LL of 23–24 days and remained relatively stable between 26.8% and 28.9% in sows with LL longer than 24 days.

Regarding LSW, the incidence of prolonged WSI increased with the number of piglets weaned, rising from 29.6% (75/253) in sows weaning fewer than 11 piglets to 40.5% (202/499) in those weaning 14 or more (Table 1).

**Table 1:** Univariate analysis of potential risk factors for prolonged weaning-to-service interval in 3,222 primiparous Landrace × Yorkshire crossbred sows.

Factors	Incidence	OR (95%CI)	p-value
AFAI (days)			
189–240	56.7 (242/427)	1	
241–250	40.8 (156/382)	0.53 (0.40–0.70)	<0.001
251–260	31.9 (183/574)	0.36 (0.28–0.46)	<0.001
261–270	28.3 (189/667)	0.30 (0.23–0.39)	<0.001
271–280	29.0 (145/500)	0.2 (0.24–0.41)	<0.001
280–436	29.6 (199/672)	0.32 (0.25–0.41)	<0.001
AFF (days)			
302–360	55.0 (298/542)	1	
361–370	35.6 (147/413)	0.45 (0.35–0.59)	<0.001
371–380	31.9 (197/618)	0.38 (0.20–0.49)	<0.001
381–390	26.4 (174/660)	0.29 (0.23–0.37)	<0.001
391–400	31.1 (127/409)	0.37 (0.28–0.48)	<0.001
400–554	29.5 (171/580)	0.34 (0.27–0.44)	<0.001
NBA			
NBA <6	31.0 (18/58)	1	
NBA=6–10	35.3 (208/569)	1.21 (0.68–2.17)	0.514
NBA=11–15	33.8 (762/2255)	1.13 (0.65–1.99)	0.661
NBA=16–21	39.1 (133/340)	1.43 (0.79–2.60)	0.243
LBW (kg)			
<10	35.5 (66/186)	1	
10–15	34.8 (362/1041)	0.97 (0.70–1.34)	0.852
15–18	32.0 (425/1328)	0.86 (0.62–1.18)	0.343
>18	39.1 (261/667)	1.17 (0.83–1.64)	0.366
LL (days)			
12–22	53.7 (376/700)	1	
23–24	34.0 (201/592)	0.44 (0.35–0.56)	< 0.001
25–26	27.5 (231/841)	0.33 (0.26–0.40)	< 0.001
27–28	28.9 (195/675)	0.35 (0.28–044)	< 0.001
>28	26.8 (111/414)	0.32 (0.24–0.41)	< 0.001
LSW (piglets)			
<11	29.6 (75/253)	1	
11	32.2 (131/407)	1.13 (0.80–1.58)	0.493
12	33.1 (361/1090)	1.18 (0.87–1.58)	0.288
13	35.5 (345/973)	1.30 (0.97–1.76)	0.083
14–18	40.5 (202/499)	1.61 (1.17–2.23)	0.004

OR=Odds ratio, 95% CI=95% confidence interval, P=Probability, AFAI=Age at first artificial insemination, AFF=Age at first farrowing, NBA=Number born alive, LBW=Litter birth weight, LL=Lactation length, LSW=Litter size at weaning

### Univariate analysis

Univariate logistic regression identified significant associations between prolonged WSI and the following factors: AFAI, AFF, LL, and LSW (p < 0.05). No significant associations were found between prolonged WSI and either NBA or LBW (p > 0.05) (Table 1).

### Multivariate analysis

The final multivariate logistic regression model included LL, AFF, and LSW as independent predictors of prolonged WSI (Table 2). The model’s explanatory power increased with the addition of each variable, as indicated by the Nagelkerke R^2^ values of 0.062, 0.090, and 0.094 for one-, two-, and three-variable models, respectively. Correspondingly, AIC values declined from 4010.857 to 3936.172, and BIC values from 4023.010 to 3960.483, indicating improved model fit.

**Table 2:** Multivariate analysis of potential risk factors for prolonged weaning-to-service interval in 3,222 primiparous Landrace×Yorkshire crossbred sows.

Factors	OR (95%CI)	p-value
LL at first parity (days)		
12–22	1	
23–24	0.50 (0.40–0.63)	<0.001
25–26	0.39 (0.32–0.49)	<0.001
27–28	0.43 (0.34–0.54)	<0.001
>28	0.39 (0.30–0.51)	<0.001
AFF (days)		
302–360	1	
361–370	0.51 (0.39–0.67)	<0.001
371–380	0.49 (0.38–0.62)	<0.001
381–390	0.38 (0.29–0.48)	<0.001
391–400	0.49 (0.37–0.65)	<0.001
400–554	0.43 (0.33–0.55)	<0.001
LSW (piglets)		
<11	1	
11	1.22 (0.85–1.74)	0.279
12	1.41 (1.04–1.93)	0.029
13	1.46 (1.07–1.99)	0.018
14–18	1.63 (1.17–2.29)	0.004

OR=Odds ratio, 95% CI=95% confidence interval, P=Probability, LL=Lactation length, AFF=Age at first farrowing, LSW=Litter size at weaning

A mild but statistically significant correlation was observed between AFF and LL (Spearman’s ρ = 0.198, p < 0.001), confirming the absence of multicollinearity. The Hosmer-Lemeshow test yielded p = 0.15, indicating good model calibration. The model’s discriminatory capacity, assessed by the AUC, was 0.736, suggesting moderate predictive ability.

AFAI was excluded from the final model due to its strong collinearity with AFF (Spearman’s ρ = 0.970, p < 0.001).

### Significant predictors of prolonged WSI

Both AFF and LL demonstrated a significant negative association with prolonged WSI:

- Sows with an AFF of 302–360 days were at greater risk of prolonged WSI compared to older age groups:
  - 361–370 days: OR = 0.51 (95% CI: 0.39–0.67, p < 0.001)
  - 371–380 days: OR = 0.49 (95% CI: 0.38–0.62, p < 0.001)
  - 381–390 days: OR = 0.38 (95% CI: 0.29–0.48, p < 0.001)
  - 391–400 days: OR = 0.49 (95% CI: 0.37–0.65, p < 0.001)
  - 400–554 days: OR = 0.43 (95% CI: 0.33–0.55, p < 0.001).
- Sows with shorter LL (12–22 days) were significantly more likely to have prolonged WSI than those with longer LL:
  - 23–24 days: OR = 0.50 (95% CI: 0.40–0.63, p < 0.001)
  - 25–26 days: OR = 0.39 (95% CI: 0.32–0.49, p < 0.001)
  - 27–28 days: OR = 0.43 (95% CI: 0.34–0.54, p < 0.001)
  - 28 days: OR = 0.39 (95% CI: 0.30–0.51, p < 0.001).

LSW showed a positive association with prolonged WSI:

- Compared to sows weaning <11 piglets:
- 12 piglets: OR = 1.41 (95% CI: 1.04–1.93, p = 0.029)
- 13 piglets: OR = 1.46 (95% CI: 1.07–1.99, p = 0.018)
- 14–18 piglets: OR = 1.63 (95% CI: 1.17–2.29, p = 0.004).

## DISCUSSION

### Comparison of WSI with previous studies

The average WSI observed in this study (8.7 days) aligns closely with prior reports on primiparous sows. Previous research has documented average WSIs of 8.5 days in Thailand [13], 10.3 days in another Thai herd [21], 8.1 days in the United States [39], and 11.34 days in Lithuanian herds [14], confirming the consistency of these findings across diverse geographic and management conditions.

### LL and its physiological implications

The inverse relationship between LL and WSI observed in this study corroborates earlier find-ings [27, 34]. One likely explanation is that extended lactation allows for improved energy balance and reduced body weight loss in primiparous sows [31, 40]. In fact, sows may begin regaining weight during days 28–35 of lactation [41], which aids in recovery from the physiological demands of lactation and prepares them for subsequent estrus [40].

Longer lactation periods may also mitigate the effects of heat stress on reproductive performance [34] and stimulate pre- and post-weaning luteinizing horm-one (LH) secretion [42]. As antral follicles exceeding 4–5 mm become increasingly LH-dependent [43], elevated LH may enhance follicular growth, increase β-estradiol concentrations in follicular fluid [44], and facilitate earlier estrus onset. The data from this study support these mechanisms, as sows with LLs longer than 22 days exhibited markedly lower WSI incidence, with the optimal benefit observed at 25–26 days, consistent with prior recommendations [45]. This finding offers a practical guideline for herd reproductive management under tropical conditions.

### Impact of LSW on WSI

A positive correlation was also evident between LSW and prolonged WSI, echoing earlier research [25, 36, 46]. Experimental studies have shown that reducing litter size late in lactation improves post-weaning repr-oductive outcomes. For example, split suckling involving only 5–7 piglets after day 21 shortened the weaning-to-estrus interval by a full day [46], and reducing the litter size to 4 piglets from day 18 to weaning reduced the interval from 5.6 to 4.6 days compared to sows with larger litters [36].

Larger LSWs contribute to more pronounced body weight loss during lactation [40], which has been linked to delayed follicular development [23] and increased WSI duration [28, 30, 31]. Thus, managing litter size near weaning may be a critical factor for optimizing post-weaning reproductive efficiency.

### Age at first insemination and farrowing as risk factors

The higher incidence of prolonged WSI among sows with an AFAI of 189–240 days and an AFF of 302–360 days suggests that younger gilts may not have attained full physical maturity at the time of first service. This aligns with the findings of Carrión-López *et al*. [7], who observed that increasing gilt body weight from <135 kg to >150 kg at first service reduced the median WSI from 5 to 4 days.

Primiparous sows must allocate energy toward fetal growth, lactation, and their own body development simultaneously [8]. Those inseminated or farrowed at a younger age likely require more nutrients for somatic growth, rendering them more susceptible to energy deficits during lactation. This nutritional stress may compromise post-weaning recovery, thereby increasing the risk of prolonged WSI.

### Lack of association with NBA and LBW

In contrast to some previous studies by Nam and Sukon [27, 32], this study found no significant relationship between NBA and prolonged WSI. A likely explanation lies in the methodological differences: While earlier studies considered all live-born piglets, the present study excluded those weighing <0.85 kg, potentially underestimating NBA and introducing a classification bias.

Similarly, LBW was not associated with WSI. This may be explained by the strong positive correlation between LBW and NBA (Spearman ρ = 0.87, p < 0.001), which could confound the independent effect of LBW on WSI duration.

## CONCLUSION

This study identified key predictors of prolonged WSI in 3,222 primiparous Landrace × Yorkshire sows managed under tropical conditions. The average WSI was 8.7 days, with 34.6% of sows experiencing a prolonged interval (>6 days). Multivariate analysis reve-aled that early AFF (302–360 days), short LL (≤22 days), and large LSW (≥12 piglets) significantly increased the risk of prolonged WSI. In contrast, LLs of 25–26 days and AFFs beyond 361 days were associated with a lower incidence of delayed post-weaning estrus.

Practical implications of these findings include refining gilt management strategies to delay breeding until adequate body maturity (e.g., AFF > 361 days), extending lactation periods to at least 25 days, and cautious management of weaning litter sizes to mitigate excessive metabolic drain. These management adjustments may contribute to reduced non-productive days and improved reproductive efficiency in tropical herd settings.

The strengths of this study lie in its large, uniform cohort of first-parity sows, the comprehensive nature of the on-farm production data, and the context-specific analysis conducted under tropical environmental conditions – an area underrepresented in the current literature.

However, several limitations must be ackn-owledged. The study was conducted on a single comm-ercial farm, which may affect external validity. Excluding data for piglets under 0.85 kg could underestimate litter parameters, and the absence of physiological indicators, such as hormonal profiles, body condition scores, or stress markers, limits mechanistic interpretations. In addition, lactational estrus – an influential but unm-easured variable – could not be factored into WSI assessments.

Future research should incorporate multi-farm datasets across diverse climatic regions and integrate physiological biomarkers, endocrine monitoring, and longitudinal reproductive tracking. Investigating genetic predispositions to prolonged WSI and evaluating the cost-benefit impact of varying LL and AFF thresholds on sow lifetime productivity would further advance this field.

This study provides novel evidence supporting the role of AFF, lactation duration, and LSW as critical determinants of prolonged WSI in primiparous sows under tropical management. These insights pro- vide practical recommendations to optimize gilt develop- ment, enhance sow reproductive performance, and improve productivity in commercial swine operations in heat-stressed regions.

## DATA AVAILABILITY

The supplementary data can be made available from the corresponding author upon request.

## AUTHORS’ CONTRIBUTIONS

NHN, DTKL, NVT, BVD, and PS: Conceived and designed the study. NHN: Collected data. NHN and PS: Analyzed data, interpreted the results, and drafted and revised the manuscript. All authors have read and approved the final manuscript.
